# Initial Outcomes From a 4-Week Follow-Up Study of the Text4baby Program in the Military Women’s Population: Randomized Controlled Trial

**DOI:** 10.2196/jmir.3297

**Published:** 2014-05-20

**Authors:** W Douglas Evans, Jasmine Wallace Bihm, Daniel Szekely, Peter Nielsen, Elizabeth Murray, Lorien Abroms, Jeremy Snider

**Affiliations:** ^1^Milken Institute School of Public HealthThe George Washington UniversityWashington, DCUnited States; ^2^Madigan Army Medical CenterDepartment of Obstetrics and GynecologyTacoma, WAUnited States; ^3^School of Public HealthUniversity of WashingtonSeattle, WAUnited States

**Keywords:** Text4baby, prenatal health care, mobile health, military health, health behavior

## Abstract

**Background:**

The use of mobile phone technologies for health promotion and disease prevention has advanced rapidly in recent years. Text4baby is a theory-based mobile health (mHealth) program in which text messages are delivered to pregnant women and new mothers to improve their health care beliefs and behaviors and improve health status and clinical outcomes. Recent evaluations of Text4baby have found that it improves targeted health attitudes and beliefs, but effects on behavior have not yet been determined.

**Objective:**

In this study, investigators aimed to evaluate Text4baby in the military women’s population.

**Methods:**

Investigators conducted a randomized controlled trial at Madigan Army Medical Center in Tacoma, Washington, from December 2011 through September 2013. All participants were pregnant women first presenting for care at Madigan. Investigators conducted a baseline assessment using a 24-item, self-administered online survey of attitudes and behaviors related to Text4baby message content. Participants were randomized to Text4baby plus usual care (intervention) or usual care alone (control). Investigators analyzed treatment effects of Text4baby on short-term targeted outcomes 4 weeks post enrollment.

**Results:**

For this study, 943 patients were randomized and completed a baseline assessment. The average patient age was 28 years and nearly 70% self-identified as Caucasian. 48.7% of enrollees (459/943) completed the first follow-up assessment. Higher rates of single and working/in-school patients dropped out of the intervention arm of the study, and we adjusted for this finding in subsequent models. However, while investigators were unable to re-survey these participants, only 1.9% of Text4baby enrollees (18/943) dropped the service during the study period. Adjusted and unadjusted logistic generalized estimating equation models were developed to assess intervention effects on measured outcomes. In the model adjusting for age, marital status, having had a previous baby, and race/ethnicity, there was a significant effect of Text4baby intervention exposure on increased agreement with belief in the importance of taking prenatal vitamins (OR 1.91, 95% CI 1.08-3.34, *P*=.024). All of these attitudes had been targeted by at least one text message during the 4-week evaluation period examined in this study. In unadjusted models, there was a significant effect of intervention exposure on belief in the importance of visiting a health care provider to be a healthy new mother (OR 1.52, 95% CI 1.01-2.31, *P*=.046) and in the health risks of alcohol during pregnancy (OR 2.06, 95% CI 1.00-4.31, *P*=.05). No behavioral effects of the intervention were observed in this analysis.

**Conclusions:**

Text4baby is a promising program that offers lessons for future mHealth activities. This large-scale study demonstrated initial effects of the program on attitudes and beliefs targeted by the messages received by women during the study period. Results confirm previous findings from Text4baby studies and other mHealth research. Future analyses will examine dosage effects of the intervention on behaviors and clinical outcomes.

## Introduction

In recent years, the use of mobile phone technology for health and health care (or mHealth) has grown rapidly as a strategy to increase patient adherence to care protocols for chronic conditions. Mobile phones allow patients to monitor and take control of their own health. A number of research studies have been conducted to test the outcomes of mobile-enabled health behavior change, drug and treatment adherence, and health care solutions, which have generally indicated that mobile solutions are effective in promoting health behaviors and health care treatment and adherence outcomes [1,2].

Despite the rapid growth of mHealth, there is relatively little research on its role as a channel through which to deliver health promotion interventions. There is also little research on the effects of mHealth exposure and dosage on outcomes [3]. Some mHealth programs have been developed as reminder systems, such as in disease management and treatment adherence programs in smoking cessation, weight loss, and diabetes self-management [4-7]. In some limited cases, mHealth approaches such as text messaging have been shown to be effective in promoting adoption of behaviors such as exercise, nutrition, family planning, and encouraging risk behavior avoidance, such as smoking cessation [6-10].

Texting, a relatively simple approach to mHealth, has been the most widely used and evaluated form of mHealth intervention [11]. Text messages can act as motivators, cues to action, and also reminder mechanisms to reinforce messages [12]. The latter use, as a reminder system to reinforce treatment adherence, is perhaps the most widely used application of text messaging as an mHealth intervention [13,14].

In the past, relatively few studies have been conducted to test the efficacy of mHealth programs as a channel for full-fledged health promotion programs. However, this situation has begun to change, one sign of which is heightened interest in mHealth theory and development of behavior change models that take the mobile phone as a delivery channel into account [5,15]. Mobile phones have unique characteristics as a channel—their ubiquity, mobility, constant availability, and multiple media modalities, among others [2,15]. The future of mHealth research is to build an evidence base that supports extensions and advancement of existing behavioral theory to address how people use mobile devices and the effects of the channel on health outcomes.

The Text4baby mHealth program [16] is an example of using text messaging for health promotion and behavior change. The service launched in February 2010 and delivers text messages to pregnant women and new mothers. The program consists of 135 distinct prenatal text messages (and a separate library of postpartum messages for new mothers, not addressed here) delivered on a schedule timed to mothers’ enrollment and due date of the baby. In this way, messages are tied to the information most needed during a particular stage of pregnancy. The program is directed by the National Healthy Mothers, Healthy Babies Coalition, and text messages and participant data are managed by Voxiva, Inc. It represents one of the largest mHealth text-based programs developed to date. Several recent studies of the Text4Baby program have found that Text4Baby changes attitudes and beliefs, but effects on behavior have not yet been established [10,17,18].

Text4baby also offers an example of the development of behavioral theory and its adaptation to the design mHealth interventions. First, following the health belief model (HBM), Text4baby messages serve as a cue to prenatal care action and behavior change targeting primarily pregnant women [19]. Second, text messaging can spread social influence and diffusion of information within a target population [20]. In Text4baby, messages diffuse risk information, such as the dangers of smoking and secondhand smoke during pregnancy and serve to reinforce other social influences, such as health care providers, friends, and family. Third, Text4baby uses social cognitive theory (SCT) to build participants’ self-efficacy and provide social models of behavior through text messaging, which develop a Text4baby brand identity and brand equity (eg, the brand’s market position, consumer loyalty) [21]. An mHealth program can serve to create a brand identity as a “friend” and “trusted source” that young women come to rely on. Messages are authentic, fun, and promote ways to live a satisfying lifestyle while reducing risk and promoting maternal and child health [17]. Audiences must develop a connection with messages and find them to be credible sources that they can rely on in order to be successful [22-24]. Future mHealth programs can build on these lessons to develop mobile health messages that address the quality of life and other motivational factors to help make recommended health behaviors fun, easy, and popular with audiences.

Over the past few years, the military has made major investments in mHealth programs to promote health and improve treatment among enlisted personnel and their families [25]. The target population, which includes active duty military personnel, spouses, and family members, face significant additional stressors beyond those in the civilian population that may affect maternal and child health outcomes. Female service members struggle to balance family and service and are much more likely to be a single parent than male troops [26]. It is estimated that about 10% of female military personnel become pregnant each year, and approximately 75,000 military children are estimated to be younger than 1 year of age [26]. Thus, there is a large Military Health Service (MHS) population that could benefit from the Text4baby program, as it is a free resource that can help support military families and address a growing demand for maternal and child health and health care services.

The current study examines the first phase of a randomized controlled trial (RCT) to evaluate Text4baby in the military women’s population at Madigan Army Medical Center (Madigan) in Tacoma, Washington*.* The first specific aim of this study was to describe the Madigan Text4baby RCT, its design, methods, and baseline (BL) data. The second specific aim was to evaluate initial outcomes of the program at 4 weeks post BL. This is also to confirm a previous smaller-scale evaluation of Text4baby conducted by the investigators in a largely Spanish-speaking immigrant population in Virginia [10].

Regarding the second aim, there were two specific hypotheses. First (H1), Text4baby will be effective in improving prenatal knowledge, attitudes, and beliefs (KAB) targeted by messages delivered during the study period. Because this was a study of short-term Text4baby effects, analysis focused on Text4baby effects on KAB targeted during the initial study period, such as taking prenatal vitamins, increased fruit and vegetable intake, visiting health care providers, and avoiding smoking or drinking. Second (H2), Text4baby will be effective in improving prenatal behaviors targeted by messages.

## Methods

### Design and Measures

The investigators conducted an RCT of Text4baby prenatal messages at Madigan Army Medical Center (Madigan), a large tertiary-care Army Medical Center in Tacoma, Washington, serving Joint Base Lewis-McCord. Female military health care beneficiaries age 18-45 years (both active duty and family members) who first presented for prenatal care at Madigan prior to 14-weeks gestation were eligible for the study. The date of pregnancy was established by last menstrual period (LMP) dating. Additional inclusion criteria included having a working cell phone and speaking and reading fluent English. Female patients meeting the inclusion/exclusion criteria were recruited for the study at the end of their initial prenatal care visit to the Madigan Obstetrics and Gynecology clinic. Following medical consultation, the health care provider asked if the patient would be willing to participate in the research study. Those who agreed met with a member of the research team in a private space in the clinic, underwent a written informed consent, and then were offered the opportunity to complete a BL survey on a password-protected computer in a private room in the clinic. Three dedicated laptops were available for participants to complete the online survey. No compensation was provided for participation.

Individuals who needed more time to decide whether to participate were allowed to leave and provide a final decision by calling or texting back. A card was provided with the name and number of Text4baby points of contact they could reach to discuss the study. Those who agreed to participate after leaving the clinic completed the BL survey online at a secure, password-protected website.

After BL survey completion, participants were assigned to a study condition (treatment or control). Using an algorithm that generated a randomized list of individual assignments to treatment or control condition for a sample of 996, investigators assigned participants either to continue with usual prenatal care (control) or receive usual care plus enroll in Text4baby (treatment). After BL, participants in both groups were surveyed again after 4 weeks. Subsequent follow-ups were conducted but are not reported here. BL data collection started in December 2011 and ended in January 2013. Follow-up data collection for the 4-week follow-up survey began in January 2012 and ended in March 2013. This study reports on these BL and 4-week follow-up data.

Participants had the option to take each of the follow-up surveys in the clinic after subsequent health care appointments or remotely, online using their own computer/device. All surveys were completed anonymously and linked by an assigned participant ID. Clinicians who met with patients were blinded—the randomization occurred outside the actual clinical visit and the trial data were not accessed by the clinicians during the study. The onsite trial coordinator responsible for data collection used the random assignment list to direct participants in the Text4baby arm to enroll in the service and was aware of participant treatment or control status.

The BL survey instrument was a 24-item Web-based tool developed by the research team that contained a battery of items drawn from previous research [10]. Variables for behavioral outcomes were derived from validated instruments, including the Behavioral Risk Factor Surveillance Survey (BRFSS) and National Health and Nutritional Examination Survey (NHANES).

The survey instrument contained a series of questions on participant attitudes, beliefs, and behaviors related to the text messages contained in Text4baby*:* nutrition, smoking, taking vitamins, alcohol use, flu shots, health care appointments, health information seeking, and related risk prevention behaviors. The instrument also captured confirmed recall, reactions, and receptivity to the text messages based on validated measures previously published [27,28]. Additionally, demographic information such as age, race, ethnicity, sponsor rank, marital status, and parity (previous live birth by the mother) was collected from the medical record. The study protocol was approved as minimal risk research by the Madigan Institutional Review Board on July 26, 2011, and served as the Institutional Review Board (IRB) of record for the George Washington University, which entered into an institutional agreement with the Department of Defense (DOD) Human Research Protection Program on August 1, 2011.

### Text4baby Intervention

During enrollment in the clinic, participants assigned to the control condition were excused immediately after completing the BL survey. Those assigned to the treatment condition were immediately directed to enroll in Text4baby by texting a phrase that tagged them as participants in the Madigan study to a designated SMS (short message service) short code to receive messages for the duration of their pregnancy (or until they dropped out of the program). This combination of enrollment phrase and short code had been established in the Voxiva Text4baby data system to identify participants as members of the Madigan evaluation study. This system ensured that only Text4baby participants who were enrolled in the Madigan study were counted in our treatment group. We monitored the control group through the Voxiva data system to ensure that none of these participants separately enrolled for Text4baby outside of our study, and none did.

The Voxiva system consists of data obtained from participants during their enrollment: mobile phone number, due date (in order to time the pregnancy text messages appropriately to the gestational age of the baby), and participant’s home zip code [29]. Voxiva did not have access to any data collected through this evaluation or any patient information stored at Madigan. Once enrolled, Text4baby participants received welcome and introductory messages and then began receiving the three text messages per week throughout their enrollment. These messages were tailored to the date of enrollment and baby’s gestational age. Thus a woman enrolling in her 10^th^ week of pregnancy would begin receiving week 10 messages.

If at any time during the pregnancy, a fetal loss occurred, patients were disenrolled from Text4baby and appropriate perinatal grief counseling was offered to the patient and her partner. Patients were also provided the option to disenroll from Text4baby by texting “STOP” from their cell phones if they no longer wanted to participate in the program.

### Sampling

The sampling frame consisted of all pregnant women who were military health care beneficiaries first presenting for initial prenatal care at Madigan (ie, had not visited another health facility for the current pregnancy). Participants were further required to be within the first 14 weeks of their baby’s gestational age. All others were excluded from the study. We drew a random sample of all women meeting criteria who first presented for care at the Madigan Obstetrics and Gynecology Clinic between December 1, 2011, and January 31, 2013. Recruitment from the sample occurred on a rolling basis, meaning that they were recruited over time until the targeted sample (as determined by statistical power requirements) was reached. Previous interventions to promote reproductive health care utilization among low- and middle-income women suggest an approximate 12% effect (intervention versus control) of such programs after a 12-month time period [30]. Power analysis estimated the required sample to be 996 participants in total; 249 participants per study condition and per stratum, assuming a 10% attrition rate at 1-year follow-up.

### Data Collection Procedures

In November 2011, before the start of data collection, the research team held an introductory meeting and training session with clinical staff at Madigan to go over the study purpose and protocol. BL surveys were generally completed in clinic, with a small number completed remotely, as previously described. Follow-up (FL) surveys were primarily completed remotely online. In an attempt to ensure high response and long-term retention, we used several methods to address noncontact and noncooperation, including (1) text messages for FL data collection notification, (2) a local phone number for participants if there were questions or a desire to schedule a time to speak with the investigators (provided a business card to participants during enrollment), (3) assurances of confidentiality, and (4) a nurse available to answer the phone and take the messages to convey to investigators. One week prior to each FL survey, the onsite research team member sent text messages reminding participants of the upcoming survey FL date and offered them the option to take the survey remotely if they would not be at the clinic. Participants were considered to have quit the study if they were unreachable after several attempts, and no further FL attempts were made. At follow-ups, the same behavioral survey instrument was repeated with the addition of a short battery of questions about exposure and reactions to the Text4baby campaign and its messages.

### Data Analysis

Stata Version 12 was used in all analyses. Descriptive statistics were calculated for all outcomes and demographic variables. Cross-tabulations of these same variables by study condition and survey time point were also calculated.

Generalized estimating equations (GEE) logistic regression was used to construct separate models for each of the attitudes, beliefs, and behavioral outcomes. Investigators estimated the odds of positive change over time in response to each of the behavioral outcome variables as a function of Text4baby text message exposure through use of an interaction term including program exposure and progression to follow-up measurement.

In addition to an unadjusted model, which strictly looked at the effect of the randomized intervention on those who completed both BL and follow-up interviews (n=459), a second adjusted model included several maternal covariates: age quintile, parity, marital status, and race. For missing data and attrition of participants, a *t* test was used to compare covariates, including sociodemographic and other variables used in the regressions, between cases with and without missing data to verify whether or not data were missing completely at random. It was determined that both maternal race and marital status, which were both missing for 22.7% (215/943) of BL participants, were potentially variables that were differentially missing for women of certain racial and marital statuses. Therefore, a multiple imputation (MI) model was constructed to account for missing race and marital status, through use of a logit function, with parity, age, and treatment status as predictors of both race and marital status [31].

The MI model also accounted for attrition through inverse probability weighting, by predicting likelihood of dropout through use of a logistical regression model, using parity, age, and imputed or actual race and marital status as predictors [32]. This regression model was then used to predict the probability of a missing case and to assign higher weights to complete cases of individuals in the sample who represent those more likely to have dropped out of the study. Sixty repetitions of the model were implemented, and results from each imputation averaged to produce point estimates for an intervention effect.

## Results

Of 1078 women who entered the Madigan OB/Gyn clinic during the study period, 996 were asked to participate, or 92.39%. Of these, 94.7% completed a BL survey online (943/996). Among the BL participants, 48.7% completed a 4-week follow-up survey (459/943).

Table 1 provides the BL sample characteristics. Overall, the sample was predominantly Caucasian (69.6%, 656/943), with an average age of 26.53 years. A majority (63.1%, 595/943) reported currently attending school or working outside of the home. Over 70% (663/943) of the participants reported being married. Close to half of the participants (47.8%, 451/943) reported having had a baby prior to this pregnancy. In terms of sponsor rank, the vast majority of participants were enlisted service members or a dependent family member of an enlisted service member (86.8%, 819/943) and the remainder commissioned or warrant officers (13.2%, 124/943).

Equivalence of means at baseline was tested by comparing the baseline treatment and control condition samples. The comparison revealed a larger, statistically significant percent reporting smoking in the last 30 days: 15.34%, (95% CI 12.08-18.58) in the control versus 9.64% (95% CI 6.95-12.32) in the treatment group, respectively. There was also a larger, statistically significant percent who reported consuming 3 or more vegetables per day in the control versus treatment group: 37.82% (95% CI 33.44-42.19) in control versus 29.98% (95% CI 25.81-34.15] in treatment, respectively.

Because of the significant loss-to-follow-up, demographic characteristics of the BL sample were compared to those who remained in the study at follow-up. That analysis revealed statistically significant differences in those reporting being married (70.31% at BL vs 76.69% at FL, *P*=.000) and in those currently working or attending school (63.10% at BL vs 53.16% at FL, *P*<.001). No differential attrition between treatment and control conditions was observed.

Table 2 presents cross-tabulations of the outcome variables by study condition and survey time point. There were significant improvements in several outcome attitudes, beliefs, and behaviors from BL to FL. However, no significant differences in improvements over time were observed between the treatment and control study conditions. Increases in strong agreement with beliefs in the importance of prenatal vitamins and health risks of drinking alcohol were observed between BL and FL. An increase in self-reported searching for health information online and consuming 3 or more serving of fruits and vegetables per day was observed. Finally, a decrease in self-reported cigarette smoking was observed between the two time points.

Table 3 summarizes the results of the GEE logistic model for the intervention effects BL to FL on measured outcomes. There was a significant treatment effect for improvement in strong agreement with the statement that “If I visit my health care provider on a regular basis, I will be a healthy new mother” in the complete-case, unadjusted model, with a greater likelihood of improvement to strong agreement in the intervention group (OR 1.52, 95% CI 1.01-2.31, *P*=.046). There was also a significant treatment effect for improvement in strong agreement with the statement that “drinking alcohol will harm the health of my developing baby” in the unadjusted model, with a greater likelihood of improvement to strong agreement in the intervention group (OR 2.06, 95% CI 1.00-4.31, *P*=.05). There were marginally significant effects, at the *P*=.10 level, for improvement in strong agreement for beliefs about the importance of eating fruits and vegetables, and in taking prenatal vitamins, to the health of the developing baby.

In the adjusted model, which accounts for four socioeconomic variables, imputations for missing values for marital status and race, and inverse probability weighting to account for the noted attrition, there were marginally significant effects for improved strong agreement with the statement “If I visit my health care provider on a regular basis, I will be a healthy new mother” as well as “Drinking alcohol will harm the health of my developing baby”. There was also a significant treatment effect for improvement in strong agreement with the statement “Taking prenatal vitamins will improve the health of my developing baby”, with a greater likelihood of improvement to strong agreement in the intervention group (OR 1.91, 95% CI 1.08-3.34, *P*=.024). There were no effects of the Text4baby intervention on any of the measured behaviors at FL.

**Table 1:** Baseline sample descriptive statistics (N=943).

Variables		n	%
**Age, years**		943	26.5
	<20	31	3.3
	20-34	837	88.8
	35+	75	7.9
**Race**			
	White	656	69.6
	Black	75	7.9
	Asian-Pacific Islander	25	2.6
	Western Hemisphere Indians	2	0.2
	Other/Unknown	185	19.6
**Ethnicity**			
	Filipino	206	21.8
	Hispanic	53	5.6
	Other Asian/Pacific Islander	19	2.0
	Southeast Asian	6	0.6
	Other/Unknown/Not Hispanic	659	69.9
**Marital status**			
	Single/Never married	72	7.6
	Married	663	70.3
	Separated/Divorced/Widowed	7	0.7
	Unknown/Null	201	21.3
**Sponsor rank**			
	Enlisted	819	86.8
	Commissioned Officers	107	11.3
	Warrant Officers	13	1.4
	Null	4	0.4
**Parity**			
	No	492	52.2
	Yes	451	47.8
**Pre-pregnancy Body Mass Index**		327	27.2
	Underweight	7	0.7
	Normal	154	16.4
	Overweight	97	10.3
	Obese	63	6.7
Ever participated in WIC Program^a^		320	33.9
Currently in school or working outside the home		595	63.1
Ever gone online to search for prenatal care information		711	75.4

^a^WIC=Nutritional Program for Women, Infants, and Children.

**Table 2:** Bivariate pre-post comparison of measured outcome variables by treatment group.

	Baseline sample (n=459)				Follow-up sample (n=459)				
Attitudes, Strongly Agree	Mean, %	95% CI	Control (n=230), %	Text4baby (n=229), %	Mean, %	95% CI	Control (n=230), %	Text4baby (n=229), %	*P* value^a^
Eating 5 or more fruits and vegetables per day is important to the health of my developing baby	68.41	63.94-72.64	70.43	66.38	67.10	62.60-71.39	64.78	69.43	.672
Taking a prenatal vitamin is important to the health of my developing baby	88.02	84.69-90.84	89.57	86.46	78.00	73.92-81.70	76.09	79.91	.000
I am prepared to be a new mother	52.94	48.26-57.58	55.22	50.66	52.07	47.39-56.72	53.49	50.66	.792
If I visit my health care provider on a regular basis, I will be a healthy new mother	48.37	43.71-53.04	50.00	46.72	44.44	39.84-49.12	40.87	48.03	.234
If I visit my health care provider on a regular basis, my baby will be healthy	44.88	40.27-49.56	43.48	46.29	42.92	38.34-47.59	39.13	46.72	.550
Smoking will harm the health of my developing baby	92.59	89.80-94.82	92.61	92.58	89.98	86.86-92.57	87.83	92.14	.161
Secondhand smoke will not harm the health of my developing baby.	18.08	14.67-21.91	18.70	17.47	18.95	15.47-22.84	18.70	19.21	.734
Drinking alcohol will harm the health of my developing baby	90.41	87.35-92.95	93.48	87.33	85.62	82.07-88.70	85.65	85.59	.025
Taking a prenatal vitamin will improve the health of my developing baby	76.25	72.09-80.07	74.78	77.73	69.50	65.06-73.68	64.78	74.24	.021
Behaviors									
Have you ever gone online to search for prenatal care information?	10.02	7.43-13.14	9.57	10.48	16.78	13.47-20.51	15.21	18.34	.003
In last 30 days, did you smoke?	11.98	9.16-15.31	15.65	8.30	4.36	2.68-6.65	6.09	2.62	.000
Since you found out about your pregnancy, have you consumed alcoholic beverages?	2.40	1.20-4.25	1.74	3.06	2.61	1.36-4.52	2.61	2.62	.833
Ate 3 or more servings of fruit a day	35.95	31.55-40.53	37.39	34.50	43.36	38.77-48.03	43.48	43.23	.022
Ate 3 or more servings of vegetables a day	32.24	27.99-36.73	33.48	31.00	33.12	28.82-37.63	30.87	35.37	.779

^a^ *P* value presented represents the difference between the baseline and follow-up sample mean.

**Table 3:** Effects of Text4baby and covariates on improvements in outcome variables from baseline to follow-up.

		Effect of intervention and time on strong agreement (unadjusted)	Effect of intervention and time on strong agreement (fully adjusted, for age [quintile], parity, imputed marital status and race, and use of inverse probability weighting to account for attrition)^a^
		(n=459)	(n=943 for baseline sample)
		OR (95% CI), *P* value	OR (95% CI), *P* value
**Attitudes**			
	Eating 5 or more fruits and vegetables per day is important to the health of my developing baby	1.49 (0.96-2.31), *P*=.075	1.47 (0.83- 2.63), *P*=.189
	Taking a prenatal vitamin is important to the health of my developing baby	1.68 (0.96-2.94), *P*=.069	1.73 (0.80-3.73), *P*=.164
	I am prepared to be a new mother	1.07 (0.673-1.57), *P*=.804	1.28 (0.74-2.23), *P*=.555
	If I visit my health care provider on a regular basis, I will be a healthy new mother	1.52 (1.01-2.31), *P*=.046	1.66 (0.98-2.81), *P*=.058
	If I visit my health care provider on a regular basis, my baby will be healthy	1.22 (0.83-1.80), *P*=.320	1.32 (0.81-2.16), *P*=.268
	Smoking will harm the health of my developing baby	1.63 (0.74-3.61), *P*=.226	2.25 (0.64-7.92), *P*=.204
	Secondhand smoke will not harm the health of my developing baby (reverse coded*)	1.14 (0.81-1.58), *P*=.450	0.82 (0.47-1.44), *P*=.491
	Drinking alcohol will harm the health of my developing baby	2.06 (1.00-4.31), *P*=.050	2.19 (0.87-5.52), *P*=.095
	Taking prenatal vitamins will improve the health of my developing baby	1.33 (0.84-2.10), *P*=.221	1.91 (1.08-3.34), *P*=.024
**Behaviors**			
	Have you ever gone online to search for prenatal care information?	0.91 (0.45-1.87), *P*=.810	0.72 (0.21-2.43), *P*=.592
	In the last 30 days, did you smoke?	0.86 (0.38-1.97), *P*=.726	1.16 (0.41-3.27), *P=*.768
	Since you found out about your pregnancy, have you consumed alcoholic beverages?	0.49 (0.15-1.61), *P*=.241	0.57 (0.14-2.36), *P*=.436
	Ate 3 or more servings of fruit a day	1.04 (0.68-1.60), *P*=.842	1.07 (0.63-1.84), *P*=.758
	Ate 3 or more servings of vegetables a day	1.29 (0.83-1.97), *P*=.249	1.17 (0.42-3.27), *P*=.795

^a^Indicates change to “strongly disagree” (reverse coded).

## Discussion

### Principal Findings

Text4baby is now a widely known mHealth program that has demonstrated the rapid scalability of behavior change interventions using the mobile phone. Since its launch in February 2010, over 670,000 individuals have enrolled in the service as of January 2014 [33]. This widespread adoption suggests that the program has broad appeal and may represent a valuable health promotion model in the area of maternal and child health [34]. Given widespread adoption, it is important to evaluate the effectiveness of such programs in changing health behaviors and affecting other health outcomes.

This pilot study examined short-term effects of Text4baby exposure 4 weeks post enrollment on attitudes, beliefs, and behaviors targeted by the text messages. Overall, we found that exposure to the text messages improved some targeted attitudes and beliefs, which partially confirms H1. Specific targeted beliefs, including those about the importance of prenatal health care, the risks of alcohol use during pregnancy, and the importance of prenatal vitamins were more likely to improve given exposure to Text4baby. These results were consistent with findings from a previous randomized controlled evaluation of Text4baby [10]. Each of these beliefs was targeted by some of the text messages delivered during the intervention period.

Thus there is substantial evidence of cognitive changes associated with Text4baby. Following the program’s theory of change [10], these changes would be hypothesized to mediate subsequent behavior changes. However, in this short-term follow-up assessment, no behavior changes were observed in this study. These results disconfirm H2; no behavioral changes were observed for any of the behavioral outcomes targeted by the text messages.

Clearly behavior change is the objective of mobile health promotion programs such as Text4baby*.* However, behavioral effects often require longer intervention periods in order to manifest [35,36]. While Text4baby was not explicitly designed on the transtheoretical model (TTM), that theory would suggest that pregnant women may be rapidly progressing through pre-contemplation to contemplation to behavioral initiation, but somewhat longer periods of time than the 4-week FL period examined in this study may be required for behavior change. As the pregnancy would generally continue for several more months, the action phase according to TTM would be more likely to occur at subsequent time points. Future studies based on longer evaluation periods may shed light on potential behavioral effects of the Text4baby program.

The implications of this study for Text4baby are further confirmation of the program’s conceptual model, published elsewhere, that is based on HBM and SCT [10]. Thus this evaluation is one step toward validating a new theoretical approach to mHealth programs—one that calls for additional research and theoretical investigation in the field. Previous communication research suggests that targeted health communications delivered using validating messaging strategies may, by themselves, have small but statistically significant effects on subsequent health cognitions and behavior [37,38]. The theory behind Text4baby, then, is that beliefs targeted by the program’s text messages will have beneficial effects on those specific beliefs, which in turn will be associated with improvements related to health behaviors. This study provides confirmation of the first association but leaves open whether the program can demonstrate behavioral effects. Future studies should also examine whether targeted beliefs that improve as a result of Text4baby exposure mediate effects on behavioral outcomes.

### Limitations

This study had two important limitations. First, there were observed differences in the BL versus follow-up samples due to attrition, with more unmarried and fewer employed or in school retained at follow-up, though not between study conditions. Single mothers and those working or in school (among the individuals who would potentially most benefit fromText4baby*)* were least likely to be retained. This may have reduced observed effects of the intervention.

Second there was higher than expected attrition from the study, at just over 50%. While very few women appear to have dropped out of Text4baby messaging, we were unable to re-contact many women. As a result, the analysis included weighting and MI procedures to account for missing data in the analyses. As a result of the attrition, statistical power was reduced as well as the ability to detect potential significant differences over time between conditions resulting in relatively wide confidence intervals for observed significant results. This limitation should be considered in light of the study’s purpose as a pilot evaluation.

### Conclusions

This study found that Text4baby participation improved attitudes and beliefs targeted by the intervention—specifically beliefs in the importance of prenatal vitamins in the adjusted model. There were also improvements in beliefs about attending health care appointments and about the importance of avoiding alcohol during pregnancy in the unadjusted model. Despite these improvements in beliefs targeted by Text4baby*,* there were no short-term effects on self-reported behavior. Future studies based on this RCT will examine whether long-term participation in Text4baby affects behaviors, and whether there is a dose-response relationship (ie, receiving more messages and/or participating for a longer period is related to outcomes). Future studies should also examine the potential for mediation effects (ie, whether targeted attitudes and beliefs mediate the relationships between exposure to Text4baby and behavioral outcomes). Finally, future studies will examine whether Text4baby participation has an effect on clinical maternal and child health outcomes.

## Abbreviations

BL: baseline
BRFSS: Behavioral Risk Factor Surveillance Survey
DOD: Department of Defense
FL: follow-up
GEE: generalized estimating equations
HBM: health belief model
KAB: prenatal knowledge, attitudes, and beliefs
LMP: last menstrual period
mHealth: mobile health
MHS: Military Health Service
MI: multiple imputation
NHANES: National Health and Nutritional Examination Survey
RCT: randomized controlled trial
SCT: social cognitive theory
TATRC: Telemedicine and Advanced Technology Research Center
TTM: transtheoretical model
